# A Mobile Phone App for Dietary Intake Assessment in Adolescents: An Evaluation Study

**DOI:** 10.2196/mhealth.4804

**Published:** 2015-11-03

**Authors:** Åsa Svensson, Christel Larsson

**Affiliations:** ^1^ Department of Food and Nutrition Umeå University Umeå Sweden; ^2^ Department of Food and Nutrition, and Sport Science University of Gothenburg Gothenburg Sweden

**Keywords:** adolescents, dietary assessment, mobile phone app, energy, SenseWear Armband

## Abstract

**Background:**

There is a great need for dietary assessment methods that suit the adolescent lifestyle and give valid intake data.

**Objective:**

To develop a mobile phone app and evaluate its ability to assess energy intake (EI) and total energy expenditure (TEE) compared with objectively measured TEE. Furthermore, to investigate the impact of factors on reporting accuracy of EI, and to compare dietary intake with a Web-based method.

**Methods:**

Participants 14 to 16 years of age were recruited from year nine in schools in Gothenburg, Sweden. In total, 81 adolescents used the mobile phone app over 1 to 6 days. TEE was measured with the SenseWear Armband (SWA) during the same or proximate days. Individual factors were assessed with a questionnaire. A total of 15 participants also recorded dietary intake using a Web-based method.

**Results:**

The mobile phone app underestimated EI by 29% on a group level (*P*<.001) compared to TEE measured with the SWA, and there was no significant correlation between EI and TEE. Accuracy of EI relative to TEE increased with a weekend day in the record (*P*=.007) and lower BMI z-score (*P*=.001). TEE assessed with the mobile phone app was 1.19 times the value of TEE measured by the SWA on a group level (*P*<.001), and the correlation between the methods was .75 (*P*<.001). Analysis of physical activity levels (PAL) from the mobile phone app stratified by gender showed that accuracy of the mobile phone app was higher among boys. EI, nutrients, and food groups assessed with the mobile phone app and Web-based method among 15 participants were not significantly different and several were significantly correlated, but strong conclusions cannot be drawn due to the low number of participants.

**Conclusions:**

By using a mobile phone dietary assessment app, on average 71% of adolescents’ EI was captured. The accuracy of reported dietary intake was higher with lower BMI z-score and if a weekend day was included in the record. The daily question in the mobile phone app about physical activity could accurately rank the participants’ TEE.

## Introduction

Limitations with traditional dietary assessment methods, such as 24-hour recall and estimated or weighed food records, are well known. Using food records to assess individual dietary intake is both time consuming and burdensome for study participants and may lead to deviations from their habitual intake [1]. Misreporting energy intake (EI), especially underestimation, is a common problem in studies where food records or other traditional dietary assessment methods are used [2]. Other problems with food records are low compliance and participation rates in dietary studies. In Sweden, a Web-based food record method was developed for use in a national dietary survey in the adult population (ie, 18 to 80 years of age) [3]. The participation rate was less than 40% and the proportion classified as underestimating EI was 16% among women and 21% among men. The highest proportion of participants underestimating EI was found in the youngest age group, 18 to 30 year olds. This proportion could have possibly been even larger if the survey had been conducted among adolescents, who may have less structured dietary habits than adults and who may be less motivated to participate in dietary surveys [4,5]. Even though assessing dietary intake among adolescents can be especially challenging, knowledge of their dietary intake is important for the study of the etiology of overweight and obesity and the outcome of interventions that are implemented to promote healthy dietary habits.

Since it is important to obtain high-quality dietary intake data, improving assessment methods is essential. Methods using technology have been developed and used in several studies [6-9]. Among these are methods using digital cameras, personal digital assistants, and mobile phones to keep food records. Methods utilizing technology for dietary assessment seem promising [10], but need to be developed for local conditions and evaluated with objective measurements. As yet there is no evaluated method which uses mobile phones to collect dietary data among adolescents in Sweden. However, the use of mobile phones is widespread among young people in Sweden; 99% of 13 to 16 year olds own a mobile phone and of these 89% have an advanced-feature mobile phone [11]. This makes it possible to introduce a dietary assessment method based on mobile phone technology. Integrating the traditional food record with technology may make the assessment of dietary intake more feasible and attractive for Swedish adolescents and thus lead to more compliance and a higher quality of data. Furthermore, using mobile phones in dietary studies also allows for additional data to be collected, for example, by querying the level of physical activity and other lifestyle habits.

Dietary assessment methods are evaluated by comparison with reference methods and one way to evaluate the accuracy of assessed EI is by comparison with total energy expenditure (TEE) [1]. The "gold standard" method of measuring TEE is the doubly labeled water (DLW) method, but since it is very expensive, less resource-consuming methods to assess TEE are needed [12]. TEE can be measured by, for example, accelerometry or by calculation of TEE from basal metabolic rate (BMR) and physical activity level (PAL). It has been shown that a mobile phone questionnaire consisting of two questions on physical activity can be used to accurately assess PAL in adult women [13]. However, a similar study has not been found conducted among adolescents. Integrating questions about PAL into a dietary assessment method may be a feasible way to evaluate reported EI without the need for additional assessment methods.

Some individuals misreport EI regardless of the dietary assessment method used [14]. When investigating groups with regard to dietary habits it is important to be aware of factors influencing the validity of collected data. Factors that could possibly affect reporting accuracy in dietary assessment are gender, age, socioeconomic position, weight, health-related behaviors, and psychological factors [14,15]. The most consistent finding is a greater underestimation among participants classified as overweight/obese compared to normal-weight individuals [14], and this has also been found to be consistent for adolescents [4].

In this study, the aim was to develop, implement, and evaluate a new mobile phone dietary assessment method regarding EI and TEE compared with objectively measured TEE with the SenseWear Armband (SWA) (TEE_SWA_). Furthermore, the aim was to investigate which individual factors among adolescents affected the reporting accuracy of EI and to compare reported EI, nutrients, and food groups against the reported intake when using a Web-based method.

## Methods

### Mobile Phone App

From 2011 to 2012, a mobile phone app was developed in collaboration with an engineering student, with the aim of obtaining a method that could be used for the assessment of dietary intake—EI (EI_app_), intake of nutrients, foods, and food groups—and TEE (TEE_app_). During the development phase, five colleagues at the Department of Food and Nutrition, and Sport Science at the University of Gothenburg, Sweden, and other departments tested the mobile phone app and gave constructive feedback. Further changes were made after the method had been used by 6 adolescents and their teacher who participated in a pilot test.

The mobile phone dietary assessment method comprises the app, which is developed for Android mobile phones, a Web project (Microsoft server), and a database (Microsoft SQL server 2008R2). The Web project is used for communication between the app and the database and to enable downloading of the app to a mobile phone. The database is used for receiving, computing, and storing participant data. The app communicates with the Web project via Wi-Fi or 3G. Participant data and results from registrations are obtained from the database using the computer software FileMaker Pro 12.0 version 3 (FileMaker, Inc, Santa Clara, CA).

To ensure that the mobile phone app used the most complete Swedish food database and that the results from the mobile phone method would be comparable to a Web-based method used in a national survey [3], information about energy and nutrient content of foods, dishes, and products—as well as portion amounts that had been used in the most recent Swedish national dietary survey—was obtained from the National Food Agency. The Swedish national food database version 2010-05-05 that is used in the mobile phone app includes over 1900 foods and dishes. Prior to use in the national survey, recipes were created for common dishes in order to facilitate the recording of these dishes. The consumed amounts are estimated with well-suited units (eg, gram, deciliter, tablespoon, teaspoon, and piece) that are given as alternatives in order to estimate portion size of each food/dish. For several items, there are also pictures of foods of known weight and increasing portion sizes to aid in the estimation of consumed amounts.

The first time the mobile phone app was used by a participant he/she was asked to register an account by entering the study ID as username and a password of their own choice. The participant also entered his/her name, date of birth, gender, weight, height, email address, and phone number. Furthermore, information was entered regarding the highest completed educational level of the mother and father (no formal education; nine-year compulsory school; two-year upper secondary school, folk high school, or vocational training; at least three-year upper secondary school; or college or university); whether the participant, mother, or father were born outside of Sweden; and whether the participant has a special diet (gluten- or lactose-free, vegetarian, or other with the opportunity to specify). Female participants were also asked to enter whether they were pregnant or breast-feeding. We regarded this information to be of interest when conducting dietary assessments and in our evaluation study. The personal settings could be edited later if necessary.

To record dietary intake, the participant first entered the date and time of a meal, with the current date and time as the default setting, and type of meal—breakfast, lunch, dinner, or snack. The participant thereafter searched for the consumed food in the food database by using a free-text search, choosing from a category or type of dish. The correct amount was entered and the next food item could be searched. After all foods in the meal had been entered, the meal needed to be saved and was automatically sent to the SQL database where energy and nutrient contents were computed and stored. The saved meals could be accessed in the mobile phone app through an archive of registered days. In the archive, foods could be deleted or added and amounts could be changed. Additional functions in the mobile phone app included receiving reminders to record meals (ie, status bar notifications) at a chosen time interval and saving a meal as a template to be loaded the next time an identical meal is consumed. The app was connected to the mobile phone camera and the user could take a picture of their meal as a memory aid if the consumed foods could not be entered until later. The additional functions were included to support the participant in recording dietary intake.

In addition to recording dietary intake during a specific day, the participant was asked to answer a few questions in the mobile phone app every evening. We included questions that were considered useful when evaluating the participant’s recorded dietary intake. The questions were about the use of nutritional supplements with some alternatives given and the option to enter other supplements. The intake of supplements was not included in the analysis in this study since supplement intake was not recorded with the Web-based method. Furthermore, the participant was asked to approximate how much of their dietary intake on the specific day was recorded in the mobile phone app, and if he/she had tried to gain or lose weight during that day. The participant was also asked to approximate his/her physical activity level for that specific day out of five predefined levels (very light, light, moderate, heavy, or very heavy), as well as if his/her dietary intake and physical activity had been higher or lower than usual. Some examples of activities were given for the different activity levels. Finally, the participant was asked whether he/she had felt stressed or anxious during registration day.

Participant feedback could be viewed per day or for a selected period of days, and some detailed feedback could be viewed for each meal. In the archive, the user could see details regarding his/her body mass index (BMI); estimated TEE; EI; and intake of macronutrients, fruits and vegetables, dietary fiber, calcium, iron, vitamin C, vitamin D, and folic acid in relation to recommended daily intakes [16]. Energy percentages of each meal in relation to recommended intakes was also given. The feedback could be viewed after sending the recorded meal to the server.

All data registered by the participant when creating an account, recording foods, or answering questions in the mobile phone app could be viewed by the researcher. Information that was saved included time stamps for the different activities in the mobile phone app and whether registered information was updated by the participant. Food groups for each recorded food were given and the amount of food was calculated in grams. Energy and nutrient content in the records could be viewed per food, meal, or day. In addition to EI, the database calculated the intake of 49 nutrients. Furthermore, PAL values and estimated TEE were calculated from the daily physical activity of the participant together with the participant’s age, gender, and weight. Basal metabolic rate was calculated using equations by Shofield [17]. PALs for adolescents were adapted from the paper by Torun, separately for girls and boys [18]. Since we wanted a wider dispersion of estimated TEE, five levels were chosen instead of three. The specific PAL values used in the mobile phone app were as follows for girls and boys, respectively: very light (1.3 and 1.4), light (1.5 and 1.6), moderate (1.7 and 1.8), heavy (1.9 and 2.0), and very heavy (2.1 and 2.2). The text describing the different activity levels were as follows: very light = sedentary most of the day; light = sedentary, standing, or walking short distances; moderate = standing or walking most of the day, *or* sedentary but with 30 to 60 minutes of walking or bicycling at moderate speed; heavy = sedentary, standing, and walking short distances, *and* 60 minutes of strenuous physical activity/sport; very heavy = standing and walking most of the day, *and* 60 minutes of strenuous physical activity/sport.

### Web-Based Method

The National Food Agency in Sweden has developed a Web-based method for dietary recording that has been used in a national survey of dietary intake [3]. The method uses the national food database version 2010-05-05 (the same as for the mobile phone method). Food intake was entered by the participant on the Internet in the evening of each day. A username and password was used by the participant to log in to a webpage, and the correct day, meal type (ie, breakfast, lunch, dinner, or other), time, and place were selected. The consumed foods/dishes were searched and entered using free-text search, choosing from a category and type of dish. The consumed amount was thereafter entered using well-suited units (eg, gram, deciliter, tablespoon, teaspoon, piece, and portion-size pictures) that were given as alternatives for each food. The participants were given a booklet with pictures of different portion sizes of known weight and a notebook in which the meal type, time and place of the meal, food, and portion size could be entered during the day in order to facilitate recording it onto the computer in the evening. The participants were thereafter able to see results for the recorded days in the form of EI (EI_Web_); nutrients; proportions of fat, protein, and carbohydrates; and amount of consumed fruit and vegetables in relation to recommendations. In this study, the participants conducted the Web-based method for 3 days.

### Energy Expenditure

The SWA Pro 2 or 3 (BodyMedia, Inc, Pittsburgh, PA, USA) was worn by the participants on the days they recorded dietary intake. The SWA is a multisensory device that estimates energy expenditure (EE) using a two-axis accelerometer set at 1-minute intervals. It also has different sensors that measure heat flux, near-body and skin temperature, and galvanic skin response. The participants were instructed to wear the SWA on the back of the upper right arm over the triceps muscle—in accordance with the manufacturer’s recommendations—on 3 full consecutive days and nights, and to remove it only when getting wet, such as when showering or swimming. When removed from the body, the SWA estimates EE as equal to the basal metabolic rate, which was calculated automatically based on the participant’s age, gender, weight, and height. The computer software SenseWear Professional version 6.1 (BodyMedia, Inc, Pittsburgh, PA, USA) was used to estimate TEE from the armband’s registrations together with information about the participant’s age, gender, weight, and height.

### Questionnaire

To obtain information about factors that could possibly influence the accuracy of the reported dietary intake, a selection of questions and questionnaire instruments were put together in a questionnaire containing 53 items that was used in this study. The questionnaire included questions about factors that have previously been found to be associated with reporting accuracy [15,19] and questions about other factors that we thought may be important (ie, conscientiousness; whether they thought what they ate was important; and whether they found the study to be comprehensible, manageable, and meaningful). The included instruments were the Three-Factor Eating Questionnaire (Revised 18-item [R-18] version) [20] and the Figure Rating Scale [21]. Furthermore, five items selected from the Brief Fear of Negative Evaluation Scale [22], and seven items selected from the Marlowe-Crowne Social Desirability Scale [23] were included, as well as 15 items assessing conscientiousness selected from the International Personality Item Pool [24]. Additional questions included those concerned with how often the participant ate lunch in the school canteen and had breakfast during a normal week; if they thought what they ate was important; and whether they perceived the study presented to them as being comprehensible, manageable, and meaningful. Indices were created from the Brief Fear of Negative Evaluation Scale, the Marlowe-Crowne Social Desirability Scale, the items assessing conscientiousness, and the three subscales in the Three-Factor Eating Questionnaire. Current and ideal body size was measured with the Figure Rating Scale and the discrepancy was categorized into no discrepancy, preferring to be smaller, or preferring to be larger.

### Anthropometric Measurements

The participants' weight and height were measured with a portable scale and stadiometer using standardized procedures. The measurements were conducted in a separate room in the school by the first author (ÅS) or an assistant. The participants were asked to take off their shoes and any heavy garment, such as a sweater, and to empty their pockets before being measured. Weight was measured to the nearest 0.1 kg and height to the nearest 0.1 cm. BMI (kg/m^2^) was calculated and BMI z-score and weight status were determined using tables and cutoffs from the International Obesity Task Force [25].

### Participants and Setting

Data collection took place in Southwest Sweden during 2013. Prior to the main study, a pilot test was conducted in November 2012 in order to test the methods and design and to practice the presentation and implementation of the study. The pilot test took place in a school outside Gothenburg, Sweden; 5 girls and 1 boy from one classroom were recruited. The participants in the pilot test used the same methods as the participants in the main study, however, they used the mobile phone app and Web-based dietary registration method on the same 3 days instead of in separate weeks. Based on the experiences from the pilot test, the methods were revised prior to the evaluation study regarding design of the mobile phone app and presentation of the study to the participants.

A sample-size analysis was performed when designing the study in order to estimate the number of participants needed to detect an 837 kJ (200 kcal) difference between EI and TEE at alpha .05 and 80% power. A 100 kcal difference between the methods could also be considered meaningful, however, this corresponds to a small amount of food such as a glass of milk or a banana, and it is not likely that a dietary assessment method will be this exact. The equation used was as follows:

n=2 × (2.8 × Standard deviation/Difference)^2^

The standard deviation was derived from a previous study among 15 year olds (n=35) in Gothenburg, Sweden, which was different between girls and boys [26]. According to the sample-size calculation, the number of girls needed was 50 and the number of boys needed was 64.

**Figure 1:**
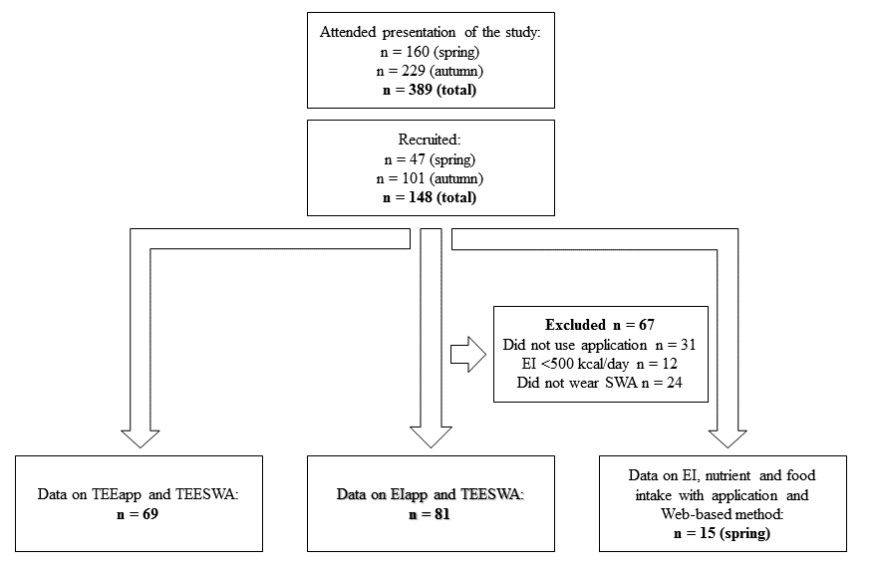
Flowchart of participants included in the different analyses.

Adolescents in year nine (14 to 16 year olds) were recruited to the study by visits to schools. Head teachers of 136 schools in Gothenburg, Sweden, and neighboring municipalities were contacted by post, email, or telephone with a short description of the study and its aims. Head teachers were asked if they would pass on email addresses or phone numbers of teachers to contact regarding the study. Teachers of physical education and health, and home and consumer studies were suggested since the study was relevant to these subjects. In some cases, contact with teachers was established through colleagues at the department, or through teachers who had been visited previously during the study year. The study was presented to teachers by email or telephone and they were asked if they were willing to assign class time to present the study and to recruit participants. Recruitment to the study was done continually throughout the year and at least two reminders were sent to all head teachers and teachers who did not respond to the first email. In total, 17 presentations of the study were held in 12 schools: five presentations in four schools during spring term and 12 presentations in eight schools during autumn term. In some cases, the study was presented only to those adolescents who had said they were interested in the study when asked by their teacher. Out of 389 adolescents from 28 school classes who were given information about the study, 148 adolescents (38.0%) were recruited, including 85 girls (57.4%) and 63 boys (42.6%) (see Figure 1).

This study was conducted according to the guidelines laid down in the Declaration of Helsinki and all procedures involving human subjects were approved by the Regional Ethical Review Board in Umeå, Sweden. Written informed consent was obtained from all participants.

### Data Collection Procedures

From January 2013 to December 2013, the first author (ÅS), together with an assistant, visited the school classes and presented the study and methods to the adolescents. They were told about the aim of the study, that participation was voluntary, and that all collected data would be treated with confidentiality. Those who chose to participate filled out an informed consent form and a questionnaire, were measured for weight and height, and were given the material needed to participate in the study. If the participant had not yet turned 15 years of age, both the adolescent and their parent or guardian signed the informed consent form. Those who did not have an Android mobile phone were given the opportunity to borrow a mobile phone with a data traffic subscription and a charger from the university, and were given an instruction manual on how to use the mobile phone (72/148, 48.6%). Those who had their own Android mobile phone were given instructions and help on how to download and install the app on their mobile phones (9/148, 6.1%). The remaining 67 adolescents were excluded. All participants were provided with an SWA and were instructed on how to use it. During spring term, participants were also given instructions and log-in details for the Web-based dietary registration method and were provided with the booklet on portion sizes and a notebook needed for using the method.

The participants were asked to complete 3 days of dietary recording using the mobile phone app. Participants recruited during the spring term were also asked to complete an additional 3 days of dietary recording using the Web-based method. Because of difficulties in recruiting adolescents and perceived high participant burden among the participants recruited during the spring term, the participants recruited during the autumn term were not asked to complete the additional 3 days of dietary recording using the Web-based method. The days for registration were decided beforehand with the aim to have Mondays to Thursdays, Fridays, and weekends equally represented in the final data. Participants who recorded dietary intake using both mobile phones and computers were asked to complete the records on the same days of week, meaning that if they recorded with the mobile phone app on Thursday to Saturday during the first week they would record using the Web-based method on Thursday to Saturday of the following week. Half of the participants were asked to start with the mobile phone app and half with the Web-based method.

All participants were asked to record everything they ate or drank during the days of registration, including snacks and condiments, and to give the consumed amounts. They were asked to eat as usual and not change their dietary intake during the study. If the participant could not find the correct food or dish in the mobile phone app then they were asked to choose a similar food or record each part of a dish one at a time, respectively. On the same days that they recorded dietary intake, the participants were instructed to wear the SWA from 00:00 (or before going to bed the night before the measurement period) to 24:00 (or until waking up in the morning after the measurement period) and to only take it off when they were going to get wet.

Group interviews were conducted with participants approximately 1 to 2 weeks after they had participated in the study in order to identify strengths and problems with the mobile phone app method. Results of the group interviews will be used in future work to improve dietary assessment methods and are not presented here.

### Data Analysis and Statistics

Statistical analyses were performed with IBM SPSS version 21, and *P* values ≤.05 were considered significant. The variables were checked for normality using the Shapiro-Wilk test. Some of the variables had a skewed distribution and, consequently, nonparametric tests were principally used.

Days with reported EI below 2092 kJ (500 kcal) were excluded, and to be included in analyses the SWA had to be worn for at least 19 hours (80%) of the day. These cutoffs were chosen in order to include a realistic completion of the methods. Implausible values of recorded food intake were found in the records of 8 participants with the mobile phone app and for 1 participant with the Web-based method. The participants in question were contacted and more realistic values were obtained.

Data are presented as median (interquartile range [IQR]), mean (SD), and percentage proportion. The Mann-Whitney U test was used to compare continuous variables between girls and boys and between participants with different weight status. Since participants classified for thinness and obesity were too few to be analyzed separately, thinness was combined with normal weight and obesity was combined with overweight in the analysis. The Wilcoxon signed rank sum test and paired-samples *t* test were used to analyze the difference between EI and TEE, as well as TEE being estimated with the mobile phone app and measured with the SWA. Bland-Altman plots and Spearman correlation coefficients were used to evaluate the reporting accuracy of EI and TEE assessed with the mobile phone app in comparison with TEE from the SWA.

In total, 15 of the 81 participants (19%) included in the main analysis had missing data on one or more questions in the questionnaire. For 2 of the participants with missing data on the Figure Rating Scale, data were imputed as *No discrepancy* between perceived and ideal body size. One participant did not reply to the question about frequency of eating breakfast and lunch in the school canteen, and for this participant the missing values were imputed with *7 days/week* and *5 days/week*, respectively. For 7 of the participants with missing data on the scale measuring conscientiousness (1-3 items each), data were imputed as the middle alternative, *Neither inaccurate nor accurate*, for the respective items. Furthermore, data were missing for the scales measuring *social desirability* (n=3, one item each), *uncontrolled eating* (n=2, one item each), and *cognitive restraint* (n=3, one, two, or three items each). These values were imputed with the median response to the items by the other participants. Results did not differ when using imputed values compared with only including data from the 66 participants with complete questionnaires.

The effect of questionnaire variables, as well as gender and BMI z-score, on reporting accuracy of EI (EI-TEE) was investigated by stepwise linear regression analysis, after having tested the variables one by one in linear regression models. The variable for eating breakfast was categorized as *7* or *less than 7 days/week*, and having lunch in the school canteen was categorized as *5* or *less than 5 days/week*. The variables regarding whether the participants found the study comprehensible, manageable, and meaningful, and whether they thought that what they ate was important were categorized as *yes* or *somewhat/no*, since few answered *no* to these questions. Additional variables in the model were school, parental education level reported by the participants (highest of both parents categorized as low, medium, or high), whether the participants and/or parent(s) were born outside of Sweden, and the presence of a weekend day in the EI record. Since there was a dependency of reporting accuracy on TEE, a second analysis was performed with the outcome variable ([EI-TEE]/TEE). Variables selected in the stepwise models were tested in a mixed linear model together with school as a random factor to take into account the dependent observations, although this did not affect the results. Factors that could possibly influence reporting accuracy were additionally investigated by calculating the mean difference between EI and TEE from the SWA, 95% CI, and Spearman correlations in subsamples based on responses to the questionnaire and questions in the mobile phone app. In these analyses, median was used as a cutoff for continuous variables.

An additional analysis was performed comparing the mobile phone app and the Web-based method of dietary assessment. Differences in EI, nutrient intakes, and food groups between the two methods were analyzed using the Wilcoxon signed rank sum test and Spearman correlations. Results from this analysis should be interpreted with caution due to the small sample size (n=15).

## Results

### Use of the Methods

The number of participants that completed the different methods are presented in Figure 1. The participants were asked to record their diet for 3 days, however, several participants recorded for up to 6 days. In total, 81 participants had assessments of both EI with the mobile phone app and TEE with the SWA. Furthermore, 58 of the participants who answered the questions in the evening in the mobile phone app also had diet data for the specific days (Figure 1). Of the 92 participants answering the questions in the mobile phone app, 69 (75%) had SWA data on the same or proximate days. Of the 47 participants expected to record their diets using the mobile phone app and the Web-based method during spring, 15 (32%) completed both methods. Because of the difficulties in recruiting participants and the low number of participants that completed all methods, we consider this to be an evaluation study rather than a validation study of the mobile phone app method.

### Participant Characteristics

Participant characteristics are presented in Table 1. The majority (69/81, 85%) of the 81 participants were classified as being of normal weight, however, 6% (5/81) were classified as thin, 14% (11/81) as overweight, and 1% (1/81) as obese. The most common response to the question about parents’ highest completed education was university or college. A majority of the participants were born in Sweden and had parents born in Sweden, but participants with parents born outside of Sweden (20/81, 25%) were also common (see Table 1). Parental education level and origin of participants did not differ between the participants and the general population from which they were recruited. A total of 4 girls and 1 boy (all normal weight) reported eating a special diet. A total of 2 girls reported a lactose-free diet, 1 girl reported a gluten-free diet, and 1 girl reported excluding red meat from her diet. The boy reported excluding pork from his diet. Data regarding factors that may influence the accuracy of reported dietary intake are presented in Table 2. In total, 12 participants out of 81 (15%) reported taking dietary supplements—multivitamins, vitamin D, vitamin C, iron, calcium, and creatine—during registration days with the mobile phone app.

**Table 1:** Characteristics of Swedish adolescent study participants.

Characteristics		All (n=81)	Girls (n=50)	Boys (n=31)	Thinness/ normal weight^a^(n=69)	Overweight/ obese^a^ (n=12)
Age in years, median (IQR^b^)		15.5 (0.5)	15.5 (0.6)	15.5 (0.4)	15.5 (0.5)	15.6 (0.5)
Weight (kg), median (IQR)		59.9 (12.5)	57.4 (9.6)^c^	66.1 (15.7)^c^	58.0 (9.8)^c^	77.2 (17.0)^c^
Height (cm), median (IQR)		170.6 (12.9)	164.9 (13.5)^c^	176.5 (12.0)^c^	170.0 (12.8)	172.8 (13.8)
BMI^d^(kg/m^2^), median (IQR)		21.1 (3.1)	21.1 (2.5)	20.9 (4.1)	20.7 (2.4)^c^	25.8 (1.4)^c^
BMI z-score^a^, median (IQR)		0.44 (0.84)	0.38 (0.71)	0.53 (1.02)	0.33 (0.76)^c^	1.73 (0.37)^c^
**Gender, n (%)**						
	Female	50 (62)	50 (100)	31 (100)	44 (64)	6 (50)
	Male	31 (38)	0 (0)	0 (0)	25 (36)	6 (50)
**Weight status** ^a^ **, n (%)**						
	Thinness grade 2	1 (1)	0 (0)	1 (3)	1 (1)	0 (0)
	Thinness grade 1	4 (5)	3 (6)	1 (3)	4 (6)	0 (0)
	Normal weight	64 (79)	41 (82)	23 (74)	64 (93)	0 (0)
	Overweight	11 (14)	6 (12)	5 (16)	0 (0)	11 (92)
	Obese	1 (1)	0 (0)	1 (3)	0 (0)	1 (8)
**Parents’ highest education** ^e^ **,** **n (%)**						
	No formal education	1 (1)	0 (0)	1 (3)	0 (0)	1 (8)
	Nine-year compulsory school	14 (17)	5 (10)	9 (29)	11 (16)	3 (25)
	Two-year upper secondary school/folk high school/ vocational training	8 (10)	5 (10)	3 (10)	6 (9)	2 (17)
	At least three-year upper secondary school	18 (22)	13 (26)	5 (16)	16 (23)	2 (17)
	College/university	40 (49)	27 (54)	13 (42)	36 (52)	4 (33)
**Born outside Sweden** ^e^ **, n (%)**						
	None	58 (72)	38 (76)	20 (65)	51 (74)	7 (58)
	Only parent(s)	20 (25)	11 (22)	9 (29)	16 (23)	4 (33)
	Only participant	1 (1)	0 (0)	1 (3)	1 (1)	0 (0)
	Participant and parent(s)	2 (2)	1 (2)	1 (3)	1 (1)	1 (8)

^a^Using cutoff values according to Cole and Lobstein [25].

^b^Interquartile range (IQR).

^c^ *P*<.001, derived from the Mann-Whitney U test of difference between girls and boys or between thinness/normal weight and overweight/obese.

^d^Body mass index (BMI).

^e^Question answered by the participant when registering as a user in the mobile phone app.

**Table 2:** Factors that may influence accuracy of reported dietary intake of Swedish 15 year olds (scale ranges for all indices are 0 to 100).

Factors		All (n=81)	Girls (n=50)	Boys (n=31)	Thinness/ normal weight^a^ (n=69)	Overweight/ obese^a^ (n=12)
Conscientiousness^b^, median (IQR^c^)		72 (23), alpha^d^=.91	70 (23)	75 (23)	68 (24)	73 (19)
Fear of negative evaluation^e^, median (IQR)		25 (25), alpha=.79	30 (25)^f^	15 (25)^f^	25 (28)	25 (24)
Social desirability^g^, median (IQR)		62 (140), alpha=.63	62 (14)	62 (19)	62 (14)	62 (22)
**Three-Factor Eating** **Questionnaire R-18** ^h^ **, median (IQR)**						
	Cognitive restraint	32 (30), alpha=.82	34 (36)^f^	18 (23)^f^	27 (32)	39 (28)
	Uncontrolled eating	33 (28), alpha=.84	33 (27)	37 (30)	33 (28)	30 (33)
	Emotional eating	11 (33), alpha=.86	22 (33)^i^	11 (22)^i^	11 (33)	11 (19)
**Figure Rating Scale, n (%)**						
	No discrepancy	25 (31)	13 (26)	12 (39)	23 (33)	2 (17)
	Prefer to be smaller	40 (49)	33 (66)	7 (22)	30 (44)	10 (83)
	Prefer to be larger	16 (20)	4 (8)	12 (39)	16 (23)	0 (0)
**Eating breakfast, n (%)**						
	7 days/week	54 (67)	32 (64)	22 (71)	47 (68)	7 (58)
	<7 days/week	27 (33)	18 (36)	9 (29)	22 (32)	5 (42)
**Lunch in school canteen, n (%)**						
	5 days/week	56 (69)	35 (70)	21 (68)	46 (67)	10 (83)
	<5 days/week	25 (31)	15 (30)	10 (32)	23 (33)	2 (7)
**Is what you eat important to you?, n (%)**						
	Yes	41 (51)	28 (56)	13 (42)	36 (52)	5 (42)
	Somewhat	34 (42)	21 (42)	13 (42)	27 (39)	7 (58)
	No	6 (7)	1 (2)	5 (16)	6 (9)	0 (0)
**Study is comprehensible, n (%)**						
	Yes	62 (77)	39 (78)	23 (74)	54 (78)	8 (67)
	Somewhat	18 (22)	10 (20)	8 (26)	14 (21)	4 (33)
	No	1 (1)	1 (2)	0 (0)	1 (1)	0 (0)
**Study is manageable, n (%)**						
	Yes	64 (79)	41 (82)	23 (74)	56 (81)	8 (67)
	Somewhat	16 (20)	8 (16)	8 (26)	12 (18)	4 (33)
	No	1 (1)	1 (2)	0 (0)	1 (1)	0 (0)
**Study is meaningful, n (%)**						
	Yes	50 (62)	32 (64)	18 (58)	42 (61)	8 (67)
	Somewhat	26 (32)	16 (32)	10 (32)	22 (32)	4 (33)
	No	5 (6)	2 (4)	3 (10)	5 (7)	0 (0)

^a^Using cutoff values according to Cole and Lobstein [25].

^b^Fifteen items selected from the International Personality Item Pool [24].

^c^Interquartile range (IQR).

^d^Cronbach alpha.

^e^Five items selected from the Brief Fear of Negative Evaluation Scale [22].

^f^ *P*<.05, derived from the Mann-Whitney U test of difference between girls and boys or between thinness/normal weight and overweight/obese.

^g^Seven items selected from the Marlowe-Crowne Social Desirability Scale [23].

^h^Revised 18-item (R-18) Three-Factor Eating Questionnaire.

^i^ *P*<.01, derived from the Mann-Whitney U test of difference between girls and boys or between thinness/normal weight and overweight/obese.

### Evaluation of Reported Energy Intake

Of the 81 participants, 10 (12%) had 1 day of assessed EI, 11 (14%) had 2 days, 54 (67%) had 3 days, and 6 (7%) had 4 to 6 days. Of the 81 participants, 13 (16%) had 1 day of measured TEE with the SWA, 17 (21%) had 2 days, 40 (49%) had 3 days, and 11 (14%) had 4 to 6 days. Of the 222 days of assessed EI, 134 (60.4%) were weekdays and 88 (39.6%) were weekend days. The number of participants out of 81 with a weekend day in their EI record was 55 (68%). Of the 222 measured days of TEE_SWA_, the number of weekdays was 145 (65.3%) and the number of weekend days was 77 (34.7%). The SWA was worn an average of 23 (SD 1) hours per day.

**Figure 2:**
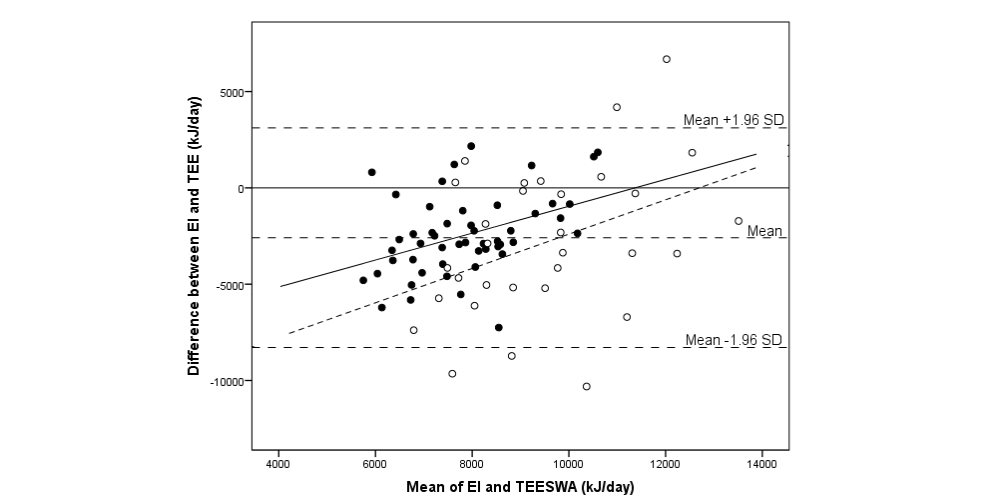
Bland-Altman plot comparing energy intake (EI) assessed with a newly developed mobile phone app and total energy expenditure measured with the SenseWear Armband (TEESWA) in 81 adolescents. The sample is displayed by gender: girls (closed circles; solid regression line) and boys (open circles; dashed regression line).

**Figure 3:**
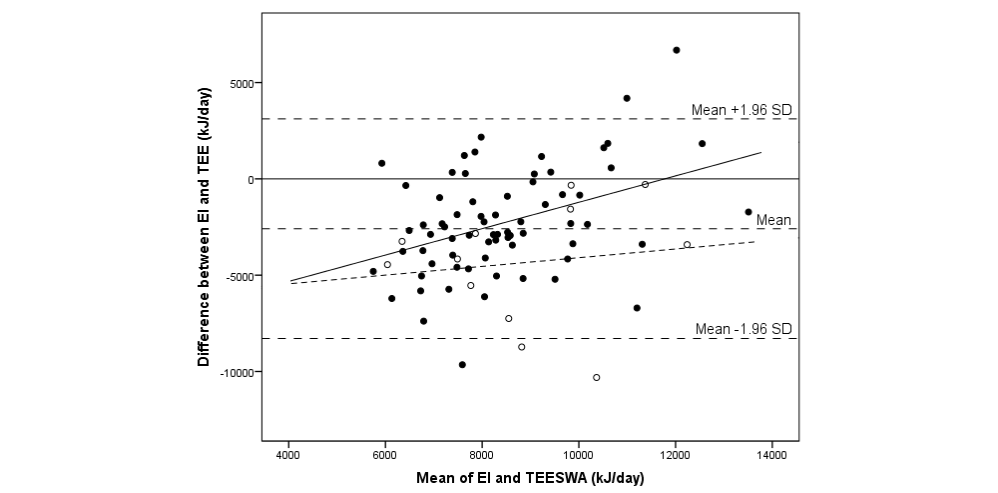
Bland-Altman plot comparing energy intake (EI) assessed with a newly developed mobile phone app and total energy expenditure measured with the SenseWear Armband (TEESWA) in 81 adolescents. The sample is displayed by weight status: thinness/normal weight (closed circles; solid regression line) and overweight/obese (open circles; dashed regression line).

**Figure 4:**
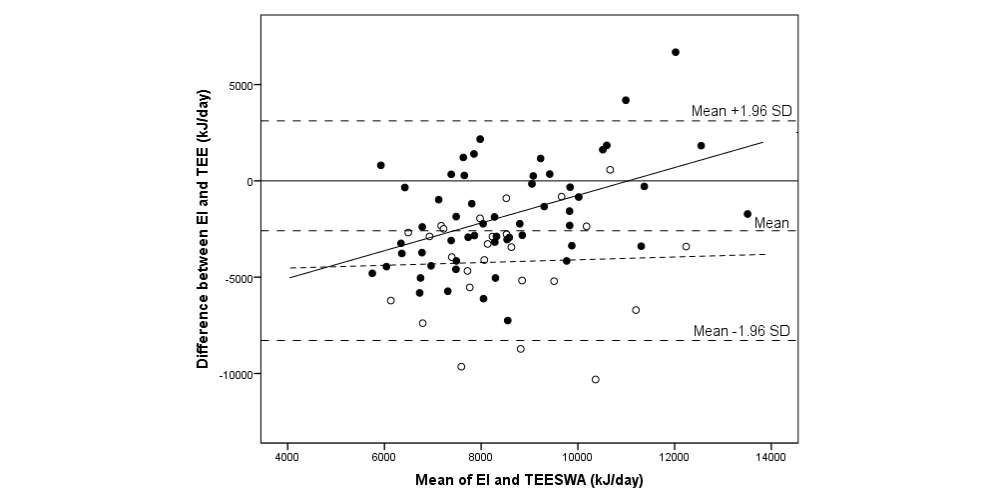
Bland-Altman plot comparing energy intake (EI) assessed with a newly developed mobile phone app and total energy expenditure measured with the SenseWear Armband (TEESWA) in 81 adolescents. The sample is displayed by records with and without a weekend day: records with a weekend day (closed circles; solid regression line) and records without a weekend day (open circles; dashed regression line).

The median difference between EI and TEE was -2837 (IQR 3452) kJ/day, and assessed EI was 71% of measured TEE by SWA (*P*<.001) (see Table 3). The corresponding result expressed as a mean difference was -2586 (SD 2908) kJ/day (95% CI -3231 to -1945), or an EI of 75% of TEE_SWA_ (*P*<.001). There was no significant correlation between EI and TEE. A total of 5 individuals out of 81 (6%)—all boys of which 1 was overweight and 1 obese—were outside the limits of agreement (see Figures 2-4). In total, out of 81 participants, 7 (9%) individuals (1 girl and 6 boys) were within ±5% of their TEE_SWA_, 63 (78%) underestimated EI, and 11 (14%) overestimated EI. Of those underestimating EI, 68% (43/63) were girls. Reporting accuracy of EI did not differ between girls and boys (Table 3).

When testing each variable, there was a positive association between reporting accuracy (EI-TEE_SWA_) and TEE (*P*<.001), and between reporting accuracy and emotional eating (*P*=.04). Reporting accuracy was higher with a weekend day in the record (*P*<.001) (Table 4). Furthermore, a negative association was found for reporting accuracy and BMI z-score (*P*<.001). In a stepwise model, TEE (*P*<.001) and a weekend day in the record (*P*=.01) were significantly associated with reporting accuracy (EI-TEE). Reporting accuracy of EI relative to TEE_SWA_, ([EI-TEE_SWA_]/TEE_SWA_), was higher with a weekend day in the record of EI (*P*=.002, *P*=.007), and lower with higher BMI z-score (*P*<.001, *P*=.001) when tested separately and in a stepwise model, respectively (Table 4). The correlation between EI and TEE was still not significant, and underestimation of EI was still significant in the analysis that included the participants (n=55) with a weekend day in the record.

Of the 58 participants who answered the daily questions in the mobile phone app, 44 (76%) stated that they had recorded 95 to 100% of their dietary intake in the mobile phone app on 87 days in total. In this sample, the median difference between EI and TEE_SWA_ was -1347 (IQR 4372) kJ/day, or EI was 88% of TEE_SWA_. When only including the 47 participants who answered that they had not felt anxious during registration day (90 days in total), the median difference between EI and TEE_SWA_ was -1753 (IQR 5134) kJ/day, or EI was 82% of TEE_SWA_. When only the 56 participants who had not tried to change their weight during registration day were included (127 days in total), the median difference between EI and TEE_SWA_ was -2004 (IQR 4263) kJ/day, or EI was 80% of TEE_SWA_. When only the 46 participants who replied that they had not felt stressed during registration day were included (91 days in total), the median difference between EI and TEE_SWA_ was -2322 (IQR 4682) kJ/day, or EI was 76% of TEE_SWA_. The correlation between EI and TEE_SWA_ was not significant in any of these subsamples and underreporting of EI was significant in all samples.

**Table 3:** Assessed energy intake and total energy expenditure using a mobile phone app, as well as total energy expenditure measured with the SenseWear Armband among Swedish 15 year olds.

Measures	All (n=81)^a^	Girls (n=50)^a^	Boys (n=31)^a^	Thinness/ normal weight^b^ (n=69)^a^	Overweight/ obese^b^ (n=12)^a^
BMR^c^(kJ/day), median (IQR^d^)	6473 (1270)	6112 (537)^e^	7641 (1161)^e^	6305 (1121)^e^	7483 (2270)^e^
EI^f^(kJ/day), median (IQR)	6904 (3427)	6527 (2410)^g^	7845 (4268)^g^	6924 (2975)	5309 (4749)
TEE_SWA_^h^(kJ/day), median (IQR)	9680 (1866)	9217 (1607)^e^	10,816 (2870)^e^	9637 (1750)^g^	10,573 (3577)^g^
TEE_app_^i^(kJ/day), median (IQR)	11,172 (2945)	10,452 (1319)^e^	13,143 (1899)^e^	10,733 (2526)^e^	13,675 (3892)^e^
EI/BMR, median (IQR)	1.08 (0.47)	1.10 (0.42)	1.06 (0.58)	1.11 (0.42)^g^	0.71 (0.56)^g^
TEE_SWA_/BMR, median (IQR)	1.48 (0.29)	1.49 (0.20)	1.36 (0.33)	1.49 (0.25)	1.36 (0.35)
EI-TEE_SWA_ (kJ/day), median (IQR)	-2837 (3452)	-2799 (2602)	-3368 (4954)	-2782 (3454)	-3787 (4937)
EI/TEE_SWA_, median (IQR)	0.71 (0.36)	0.70 (0.28)	0.74 (0.49)	0.72 (0.36)	0.58 (0.41)
TEE_app_-TEE_SWA_ (kJ/day), median (IQR)	1683 (1696)	1524 (944)^j^	2516 (1897)^j^	1575 (1480)^g^	2358 (2307)^g^
TEE_app_/TEE_SWA_, median (IQR)	1.19 (0.17)	1.17 (0.13)	1.23 (0.26)	1.18 (0.18)	1.21 (0.30)
Correlation of EI and TEE_SWA_, ρ (*P*)	.13 (.24)	.27 (.06)	-.16 (.39)	.14 (.27)	.27 (.40)
Correlation of TEE_app_ and TEE_SWA_, ρ (*P*)	.75 (<.001)	.59 (<.001)	.69 (<.001)	.74 (<.001)	.59 (.04)

^a^In the analysis comparing total energy expenditure assessed with mobile phone app and total energy expenditure from the SenseWear Armband, n=69: 41 girls and 28 boys, 57 thinness/normal weight and 12 overweight/obese.

^b^Using cutoff values according to Cole and Lobstein [25].

^c^Basal metabolic rate (BMR) calculated according to Shofield [17].

^d^Interquartile range (IQR).

^e^ *P*<.001, derived from the Mann-Whitney U test of difference between girls and boys or between thinness/normal weight and overweight/obese.

^f^Energy intake (EI).

^g^ *P*<.05, derived from the Mann-Whitney U test of difference between girls and boys or between thinness/normal weight and overweight/obese.

^h^Total energy expenditure measured by the SenseWear Armband (TEE_SWA_).

^i^Total energy expenditure reported via the mobile phone app (TEE_app_).

^j^ *P*<.01, derived from the Mann-Whitney U test of difference between girls and boys or between thinness/normal weight and overweight/obese.

**Table 4:** Factors influencing reporting accuracy of energy intake.

Factors		Univariable model (EI^a^-TEE^b,c^), *b* (95% CI)	Stepwise multivariable model (EI-TEE^c^), *b* (95% CI)	Univariable model (EI-TEE)/TEE^c^, *b* (95% CI)	Stepwise multivariable model (EI-TEE)/TEE^c^, *b* (95% CI)
**Gender**					
	Girl	N/A^d^		N/A	
	Boy	-367 (-1696, 962)		0.04 (-0.09, 0.16)	
Body mass index z-score^e^		-1509 (-2206, -812)^f^		-0.13 (-0.20, -0.07)^f^	-0.12 (-0.18, -0.05)^g^
TEE^c^(kJ)		-0.82 (-1.13, -0.51)^f^	-0.70 (-1.01, -0.39)^f^	N/A	N/A
**Parents’ education** ^h^					
	Low	462 (-2357, 1433)		-0.02 (-0.20, 0.16)	
	Medium	N/A		N/A	
	High	195 (-1277, 1668)		-0.001 (-0.140, 0.140)	
**Born outside Sweden** ^h^					
	No	N/A		N/A	
	Participant and/or parent(s)	-1020 (-2438, 397)		-0.11 (-0.25, 0.03)	
**Weekend day in record**					
	No	N/A	N/A	N/A	N/A
	Yes	2380 (1099, 3660)^f^	1557 (351, 2763)^g^	0.20 (0.08, 0.33)^g^	0.16 (0.05, 0.28)^g^
**School**					
	School 1	N/A		N/A	
	School 2	755 (-2375, 3884)		0.08 (-0.22, 0.38)	
	School 3	-683 (-3930, 2564)		-0.05 (-0.36, 0.26)	
	School 4	-1165 (-4412, 2082)		-0.11 (-0.43, 0.20)	
	School 5	-2108 (-5238, 1021)		-0.12 (-0.42, 0.18)	
	School 6	-1323 (-4571, 1924)		-0.13 (-0.45, 0.18)	
	School 7	-403 (-3180, 2372)		0.02 (-0.25, 0.29)	
	School 8	2337 (-911, 5584)		0.26 (-0.05, 0.58)	
	School 9	106 (-3300, 3512)		0.005 (-0.32, 0.33)	
	School 10	2164 (-965, 5293)		0.23 (-0.08, 0.53)	
	School 11	242 (-2887, 3371)		0.03 (-0.27, 0.33)	
	School 12	-942 (-4348, 2464)		-0.09 (-0.42, 0.24)	
Fear of negative evaluation		15 (-23, 53)		0.001 (-0.002, 0.005)	
Conscientiousness		27 (-14, 67)		0.002 (-0.002, 0.006)	
Social desirability		6 (-46, 57)		-0.001 (-0.005, 0.005)	
Cognitive restraint		-9 (-40, 23)		-0.002 (-0.005, 0.001)	
Uncontrolled eating		7 (-26, 41)		0.001 (-0.002, 0.004)	
Emotional eating		28 (2, 55)^i^		0.002 (-0.001, 0.005)	
**Prefer to be**					
	Smaller	425 (-1047, 1897)		0.04 (-0.11, 0.17)	
	No discrepancy	N/A		N/A	
	Larger	1445 (-403, 3294)		0.170 (-0.002, 0.350)	
**Breakfast**					
	<7 days/week	N/A		N/A	
	7 days/week	141 (-1232, 1515)		0.006 (-0.130, 0.140)	
**Lunch in school canteen**					
	<5 days/week	N/A		N/A	
	5 days/week	-974 (-2358, 411)		-0.11 (-0.24, 0.02)	
**What I eat is important**					
	Somewhat/no	N/A		N/A	
	Yes	350 (-942, 1643)		0.02 (-0.10, 0.15)	
**Study is comprehensible**					
	Somewhat/no	N/A		N/A	
	Yes	468 (-1056, 1993)		0.01 (-0.14, 0.16)	
**Study is manageable**					
	Somewhat/no	N/A		N/A	
	Yes	1363 (-198, 2923)		0.10 (-0.05, 0.25)	
**Study is meaningful**					
	Somewhat/no	N/A		N/A	
	Yes	1030 (-282, 2341)		0.07 (-0.06, 0.20)	

^a^Energy intake (EI).

^b^Total energy expenditure (TEE).

^c^Total energy expenditure measured by SenseWear Armband (TEE_SWA_).

^d^Not applicable (N/A).

^e^According to Cole and Lobstein [25].

^f^ *P*<.001.

^g^ *P*<.01.

^h^Question answered by the participant when registering as a user in the mobile phone app.

^i^ *P*<.05.

### Evaluation of Reported Total Energy Expenditure

Of the 69 participants included in this analysis, 17 (25%) had 1 day of TEE estimated with the mobile phone app, 18 (26%) had 2 days, 26 (38%) had 3 days, and 8 (12%) had 4 to 5 days. Of the 69 participants, 11 (16%) had 1 day of TEE measured with the SWA, 16 (23%) had 2 days, 34 (49%) had 3 days, and 8 (12%) had 4 to 6 days.

**Figure 5:**
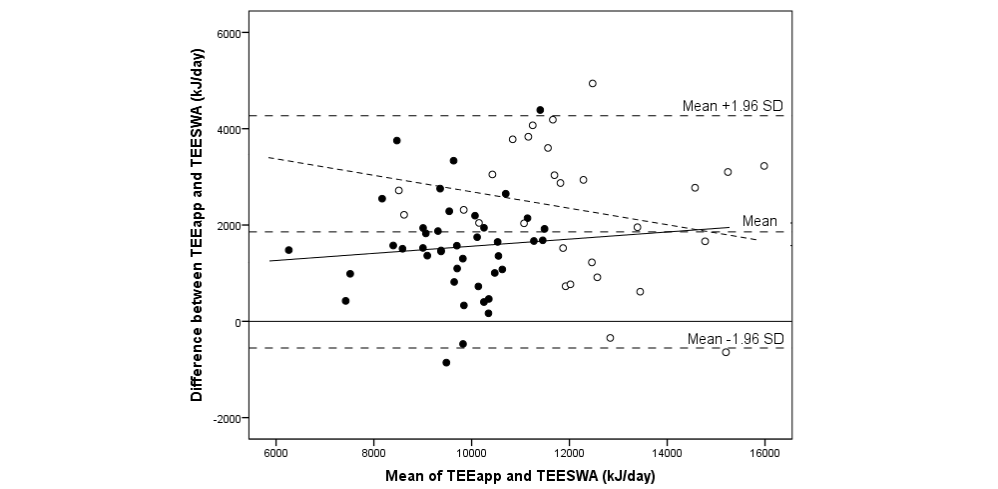
Bland-Altman plot comparing total energy expenditure estimated with a newly developed mobile phone app (TEEapp) and with the SenseWear Armband (TEESWA) in 69 adolescents. The sample is displayed by gender: girls (closed circles; solid regression line) and boys (open circles; dashed regression line).

**Figure 6:**
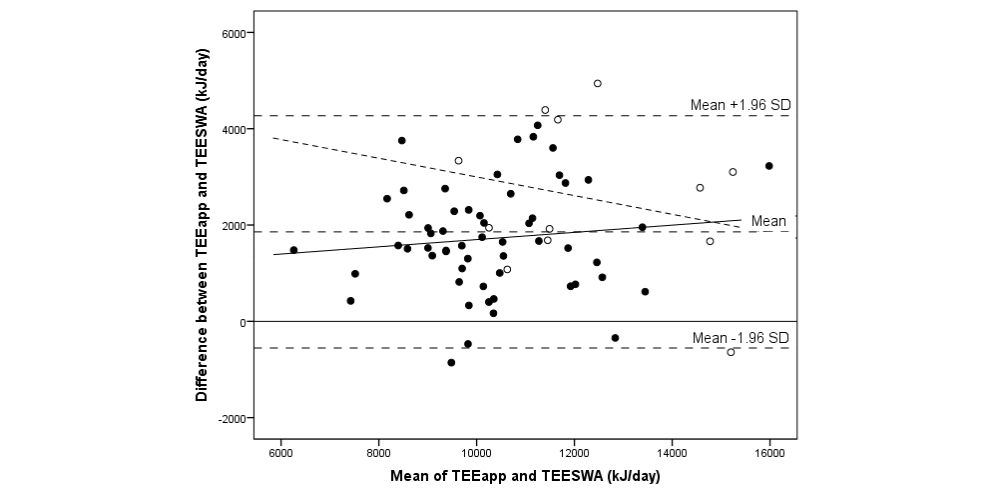
Bland-Altman plot comparing total energy expenditure estimated with a newly developed mobile phone app (TEEapp) and with the SenseWear Armband (TEESWA) in 69 adolescents. The sample is displayed by weight status: thinness/normal weight (closed circles; solid regression line) and overweight/obese (open circles; dashed regression line).

The median difference between TEE_app_ and TEE_SWA_ was 1683 (IQR 1696) kJ/day, or TEE_app_ was 1.19 times the value of TEE_SWA_ (*P*<.001) (see Table 3). The corresponding result expressed as mean difference was 1858 (SD 1230) kJ/day (95% CI 1563-2153), or TEE_app_ was 1.20 times the value of TEE_SWA_ (*P*<.001). A total of 4 individuals out of 69 (6%) were outside the limits of agreement: 2 boys and 2 girls, of which 1 was normal weight (see Figures 5 and 6). In total, 8 individuals out of 69 (12%)—5 girls and 3 boys—were within ±5% of measured TEE_SWA_. Underestimation of TEE_app_ was seen in 1 (1%) participant out of 69, and overestimation was seen in 60 (87%) participants. The correlation between TEE from the mobile phone app and the SWA was .75 (*P*<.001). A correlation coefficient of .63 (*P*<.001) was seen when comparing participants' weight with TEE_SWA_. However, PAL values derived from the mobile phone app were also significantly correlated with TEE_SWA_ (.37, *P*=.002) and with average metabolic equivalents from the SWA (.31, *P*=.009). Analysis by gender showed that correlations of PAL values from the mobile phone app with TEE (.49, *P*=.008) and average metabolic equivalents (.54, *P*=.003) from the SWA were statistically significant only for boys.

### Comparison Between Mobile Phone App and Web-Based Method

Of the 15 participants included in this analysis, 1 individual (7%) used the mobile phone app to register diet on 1 day, 3 (20%) used it on 2 days, and 11 (73%) used it on 3 days. The Web-based method was used by 3 participants (20%) on 2 days and by 12 participants (80%) on 3 days. Of the 40 days with dietary data from the mobile phone app, 29 (73%) were weekdays and 11 (28%) were weekend days. Of the 42 days with dietary data from the Web-based method, 30 (71%) were weekdays and 12 (29%) weekend days.

The median EI assessed with the mobile phone app was 6011 (IQR 4072) kJ/day and with the Web-based method was 6899 (IQR 2579) kJ/day (*P*=.36). The correlation between EI assessed with the mobile phone app and the Web-based method was .53 (*P*=.04). Of the macronutrients, fat (.54, *P*=.04), fiber (.60, *P*=.02), and monosaccharides (.67, *P*=.007) were significantly correlated between the mobile phone app and the Web-based method. Of the 18 investigated micronutrients, folate (.59, *P*=.02), iron (.66, *P*=.008), vitamin A (.55, *P*=.04), and vitamin E (.75, *P*=.001) were significantly correlated between the methods. Furthermore, six of the 34 investigated food groups were significantly correlated: beer, wine, and spirits; breakfast cereals; milk, fermented milk, and yogurt; nuts and savory snacks; soft drinks, sport drinks, and energy drinks; and sugar, syrup, honey, and artificial sweeteners (results not shown). Intake of protein, juice, and pasta were significantly different when the two methods were compared. The correlation between EI_app_-TEE and EI_Web_-TEE was .74 (*P*=.002), indicating a similar reporting accuracy of participants with the two methods. Reporting accuracy of EI compared with TEE from the SWA did not differ significantly between the methods.

## Discussion

### Principal Findings

The results showed that a mobile phone app for the recording of dietary intake captured a median EI that was 71% of TEE from the SWA in adolescents, and that there was no correlation between EI and TEE. Furthermore, BMI z-score and the presence of a weekend day in the EI record were the only investigated variables associated with reporting accuracy relative to TEE. Reported EI was almost 90% of TEE when only including the participants who said that they had recorded almost all of their dietary intake. EI, nutrients, and food groups assessed with the mobile phone app and Web-based method were generally not significantly correlated and not significantly different. TEE assessed with the mobile phone app was 1.19 times the value of TEE from the SWA on a group level, and there was a significant correlation between the two methods for TEE, and for physical activity among boys.

In a systematic review from 2010, the authors concluded that adolescents underestimate EI with food records by 18 to 42% [27], which is in line with the results of this study. A mobile phone food record method has been developed for use among adolescents, in which food images taken with the mobile phone camera are automatically identified and quantified, and nutrients are calculated [28]. To our knowledge, there are as yet no studies among adolescents evaluating assessed intakes from mobile phone food records with reference methods in free-living conditions. A previous study in 1998 among Swedish adolescents showed a mean reported EI for boys and girls of 82% and 78% of TEE, respectively, using a traditional pencil-and-paper food record over 7 days [29]. A study on US adolescents showed that the mean reported EI with a traditional food record over 2 weeks was 81% and 59% of TEE in nonobese and obese participants, respectively [30].

Factors influencing the accuracy of reported EI among adolescents have previously been described as belonging to the categories anthropometric, sociodemographic, psychosocial, behavioral, and parental characteristics [31]. In a sample of 11- to 17-year-old French adolescents, variables positively associated with underestimating EI included being overweight, having a wish to weigh less, having a restrictive diet, eating breakfast less than 7 days per week, and irregular school canteen attendance when tested individually in logistic regression models [15]. In a stepwise model, for example, being overweight and having a wish to weigh less were significantly associated with underestimation in the same study. In this study, wishing to weigh less, having a restrictive diet, eating breakfast less than 7 days per week, and irregular school canteen attendance were not associated with an underestimation of EI. Since reporting accuracy showed a dependence on the average energy values, the analysis was performed with the outcome variable, (EI-TEE)/TEE. BMI z-score and having a weekend day in the record of EI were the only investigated variables significantly associated with reporting accuracy relative to TEE. Including a weekend day in the record of EI has previously been shown to increase reporting accuracy in a sample of overweight and obese Swedish children who kept food records with the help of digital cameras [7]. One explanation could be that the participants have more time during the weekend to complete a food record. Another possible explanation is that participants could have a higher EI during the weekends and that this is reflected in the diet records. However, previous studies in Swedish children have shown that EI was not significantly different between days of the week [32-34]. The negative association between BMI and reporting accuracy has been shown previously in Swedish 15 year olds [29].

Bexelius et al constructed a mobile phone-distributed questionnaire consisting of only two questions about physical activity which adult women replied to every day for 2 weeks [13]. Aggregated PAL values were in good agreement with PAL from the DLW method, although within-subject variation in PAL between different days was high. Further evaluating the daily PAL compared with PAL from accelerometry showed that there was a true high within-subject variability in activity levels [35]. In this study, using only one question about the activity level at the end of each day gave TEE that correlated well with TEE from the SWA, even with only a few days use of each method. Since both the SWA and the mobile phone app use the participant’s weight to calculate TEE, a strong correlation between the methods can be expected. A statistically significant correlation was obtained when comparing TEE from the SWA with participants’ weight. Statistically significant correlations were, however, also found when comparing PAL values from the mobile phone app with TEE and the average metabolic equivalents from the SWA, but only for boys. Furthermore, TEE from the mobile phone app was overestimated compared with the SWA, indicating that the PAL values were set too high and should be moderately adjusted.

The analysis comparing EI, nutrients, and foods between the mobile phone app and the Web-based method included only 15 individuals and the two methods were used during separate weeks. Since dietary intake varies from day to day, it cannot be expected that the same foods are captured with the two methods. We considered the option of having both methods conducted on the same days. However, this involves drawbacks such as increased respondent burden and the methods influencing each other. It was therefore decided to conduct the methods on separate weeks but on the same days of the week. When investigating the relative validity of a method it is desirable to use a reference method with independent errors, for example, comparing food records with 24-hour recall. This study compared two different food records which, since they have the same embedded errors, could lead to an overestimation of the correlation between the methods. Monosaccharides, folate, iron, vitamin A, and vitamin E were the only nutrients significantly correlated between the two methods. Furthermore, six of the 34 food groups were significantly correlated, and one nutrient and two food groups were significantly different between the methods. These results are most likely attributable to the small sample size in this analysis. It should also be kept in mind that the many statistical tests increase the risk of chance findings.

It proved to be difficult to recruit participants to this study. Of the 389 invited adolescents, 38.0% (148/389) decided to participate. Of these, 54.7% (81/148) completed the mobile phone food record and the SWA and were included in the main analysis (81/389, 20.8% of those invited). The poor participation rate reduces the power to detect differences. Furthermore, this study includes a selection process for participants which was done in several steps. It can be assumed that a certain category of teachers agreed to assign class time for the study, and that the adolescents who decided to participate differed in motivation from those who did not.

Previous research has shown that practice with the equipment makes the participants more skilled in using it [36]. It is, however, not always possible to give the participants training prior to a dietary study. We aimed to make a mobile phone app that was self-explanatory, and handed out instructions to the participants. Despite this, giving the participants more training with the mobile phone app might have made the recording of dietary intake easier for them. Furthermore, most participants borrowed an Android mobile phone from the university to use in the study. Being unaccustomed to the type of mobile phone used could have also affected the reporting of dietary intake by making it more difficult for the participants. The participants received feedback about their registered dietary intake. They were asked not to change their intake based on the feedback; however, they might have done so.

### Limitations

Limitations of this study include the few days of registration, since EI varies over time. Some of the participants had only 1 day of recorded EI, however, 60% had 3 days or more. The proportion of weekends in the data from the mobile phone app and the SWA were approximately the same. The SWA may not have accurately measured the TEE of the participants. We have not been able to find validation studies of the SWA in measuring TEE among adolescents, and studies conducted in other groups show varying results [37]. In a validation study of the SWA against DLW in overweight children aged 8 to 11 years where the software programs Innerview Professional 5.1 and SenseWear Professional 6.0 were evaluated, valid results on a group level were shown for Innerview Professional 5.1 but not for SenseWear Professional 6.0 in this age group [38]. Furthermore, the SWA was not valid on an individual level. Although we do not know whether the same results apply to our age group, the fact that EI from the mobile phone app and TEE from the SWA did not significantly correlate in this study may be due to the SWA not being valid to measure TEE on an individual level.

### Conclusions

In conclusion, the mobile phone food record app did not accurately assess EI of adolescents when compared with TEE from the SWA in this evaluation study. Having a weekend day in the record of EI improved reporting accuracy, and BMI z-score was negatively associated with reporting accuracy. Furthermore, the mobile phone app was able to accurately rank adolescents’ TEE, as well as the physical activity level among boys by using only one question about physical activity at the end of the day.

Further development of the mobile phone app method should focus on improved functions to search and record consumed foods, for example, by automatizing these steps as much as possible. Users could, for example, have the option of sending food photographs to the researcher. The app should also be developed for iPhone so that more participants will be able to use their own mobile phones.

## Abbreviations

BMI: body mass index
BMR: basal metabolic rate
DLW: doubly labeled water
EE: energy expenditure
EI: energy intake
EI_app_: energy intake reported via the mobile phone app
EI_Web_: energy intake recorded using the Web-based method
IQR: interquartile range
N/A: not applicable
PAL: physical activity level
R-18: Revised 18-item (Three-Factor Eating Questionnaire)
SWA: SenseWear Armband
TEE: total energy expenditure
TEE_app_: total energy expenditure reported via the mobile phone app
TEE_SWA_: total energy expenditure measured by the SenseWear Armband
